# Health care benefits package design to improve outcomes in resource-constrained settings: suggestions for Tajikistan

**DOI:** 10.3389/frhs.2025.1617679

**Published:** 2025-09-16

**Authors:** Jens Wilkens, Alona Goroshko, Malika Khakimova, Farrukh Egamov, Triin Habicht, Ilker Dastan

**Affiliations:** ^1^ Department of Clinical Sciences, Lund University, Malmö, Sweden; ^2^ WHO Country Office, Dushanbe, Tajikistan; ^3^ World Health Organization Barcelona Office for Health Systems Financing, Barcelona, Spain

**Keywords:** benefits package, prioritization, universal health coverage, effective primary health care, budget resources

## Abstract

The Tajik health system is characterized by improving health outcomes over the last 20 years, but also major inefficiencies in health care delivery and large difficulties for people to access affordable essential health care. The benefits package (BP) is a key tool for prioritizing state budget allocations toward health interventions that most effectively enhance health system performance. This article suggests directions for a new BP, which is envisioned in the currently ongoing reforms. Using a universal health coverage framework, the article examines how the public budget is prioritized in the current BP and investigates the main performance challenges addressed in the government's ongoing health reform efforts from a BP perspective. Criteria for a new BP are outlined, along with suggestions for their application. The article explains how BP design can improve health system performance in a budget-constrained setting by focusing on primary health care interventions and eliminating user fees for child and maternal health services, without requiring additional budget resources. The main strength of the current BP is the provision of free family doctor and nurse consultations for all. However, its narrow scope and high user fees for essential interventions hinder both the effective management of highly prevalent chronic non-communicable diseases and the efficient use of public resources. The user fee exemptions for vulnerable population groups are neither rational in design nor possible to evaluate due to the absence of data.

## 1 Introduction

Developing a publicly funded defined health care benefits package (BP) is a key prioritization tool in health systems. Clarity over which interventions are publicly funded and which involve cost-sharing help to focus resources toward the best possible outcomes (1).

In the Republic of Tajikistan, the current BP was approved in 2008. Since then, regulations have been added and alternative descriptions have been practiced in selected geographical areas but with marginal differences (2). From its inception, the BP has had important and helpful features, such as providing free family doctor consultations and maternal and child health services. But it falls short in effectively managing key chronic conditions due to the narrow scope of interventions covered and by not funding basic laboratory services and essential medicines. It also includes high formal user-fees for specialized services and hospital treatment. Furthermore, implementation has never been aligned with the budget process, which continues to allocate resources through traditional budget lines without specifying the health problems addressed or the interventions funded. The Government's *Strategy on Healthcare of the Population of the Republic of Tajikistan for the Period to 2030* describes health sector challenges and development objectives. It emphasizes the need to solve the large and growing prevalence of chronic conditions like hypertension and diabetes, improve maternal and child health, raise the health impact of public spending, and increase the financial protection for people in need of care. The currently commencing Sughd Oblast health financing pilot (3) and the World Bank (WB) funded *Millati Solim* program (4), both building on a contractual relationship design between government entities and health providers, need the interventions that are to be funded to be defined in order for the projects to work as intended. The Government of Tajikistan is therefore currently revising the BP to better serve new budgeting and purchasing arrangements and ultimately make better use of health budget resources.

This article presents key benefit design directions for a new generation of the BP in Tajikistan to meet the country's stated health sector objectives, based on global evidence and best practices, and thereby support the implementation of current reform efforts. he descriptive analysis leading to these suggestions begins with an assessment of the current BP using a conventional universal health coverage (UHC) framework, considering three dimensions: population, interventions, and cost sharing. (5). Guided by the objectives in the country's health strategy, key challenges with the current BP are described. The suggested directions for a new BP in Tajikistan are then presented, based on a criteria approach to prioritization, as presented by the WHO and practiced in many countries (6). Examples of phased implementation from neighboring countries with a similar context and an estimation of budget effects of a new user-fee structure are used for implementation guidance. This article meets a gap in the global literature, particularly in central Asian countries, on how to apply UHC principles for the design of benefit systems.

The term BP refers to the health interventions on which government health sector resources should be spent, including health care services, medical products, or combinations of the two.” The *current* BP refers to the Government's decree No. 600, which should not be confused with an alternative, although similar, benefit description by the name *State Guaranteed Benefit Package*, which was used in parts of the country until 2023 (7).

## 2 The current benefits package

### 2.1 Population coverage

The population coverage dimension of the BP is defined by citizenship (8). Tajikistan has approximately one million people residing outside the country for work (9). By ensuring coverage for all people, as compared to a residency- or contributory-based entitlements system, from which they would have been excluded, this definition is an inclusive, transparent, and administratively efficient approach.

### 2.2 Intervention coverage

At its independence in 1991, Tajikistan inherited a health system in which prioritization of resources was absent, as all available health care was free. In several steps over the course of 20 years, the Ministry of Health and Social Protection (MoHSP) has developed the benefits description in several steps (2). Aligned with primary health care (PHC) reforms, Tajikistan formally established free point-of-care services for the most essential health care, including a broad and general description of doctor and nurse appointments, with priority for maternal and child care. The explicitly described universal benefits in the current BP include a range of preventative services (such as vaccinations for children, consultations on healthy lifestyle, and disease prevention activities) as well as consultations with family doctors and home visits by nurses (Supplementary File 1). The BP further describes a long range of additional services, both outpatient and inpatient, but these are all associated with heavy user-fees and a complicated exemptions system (8). Medicines, with vaguely regulated and very large differences in prices and availability among retailers (10), are absent in the BP. By tradition, PHC facilities do distribute some medicines, such as insulin, although with varying availability. Laboratory services are formally included for defined vulnerable groups but data on utilization are not available.

### 2.3 Cost-sharing

All interventions that are not provided by the family doctor or nurse come with a user-fee based on a nationally estimated cost. The national hospital price list is regulated centrally and is based on an 80% cost-sharing principle, conditional on the hospitalization occurring at the facility where the patient is registered (determined by place of residence) and subject to a doctor’s referral. All other utilization, including outpatient services, is priced at 100% of the cost. An important aspect is that the estimated costs are probably below actual provider costs, which makes these formal percentages lower in reality. Laboratory services and medicines for the general population are fully funded by patients themselves (8).

The BP defines vulnerable population groups who are formally exempted from user-fees (“beneficiaries”) using two dimensions: social status and a set of medical conditions. The largest beneficiary group is individuals defined by their poverty status. But the social register used to identify these households classify only 1.6% of the total population as poor, despite 23% of the population in 2021 falling under the national poverty line (11). There is no health information system to monitor who is granted beneficiary status, to what extent they overlap, nor what their care utilization is.

## 3 Key challenges with the current BP

### 3.1 Ineffective primary health care

Chronic non-communicable diseases (NCDs) like hypertension and diabetes constitute a large and growing part of the disease burden in Tajikistan (12). These conditions are manageable at low cost in PHC, especially if managed early**.** Official statistics underestimate them as indicated by the much lower prevalence in the official records than in survey-based data. The latter data also show low patient awareness and severe under-treatment, e.g., that 44% of men have never had their blood pressure measured (13, 14). It has been shown that many hospital cases in Tajikistan could be prevented if these risk-factors and conditions were managed better (15). Unnecessary hospitalizations resulting from ineffective PHC are a major factor in the estimate that half of all hospital spending in Tajikistan could be saved without adverse effects for patients (16).

**Figure 1:**
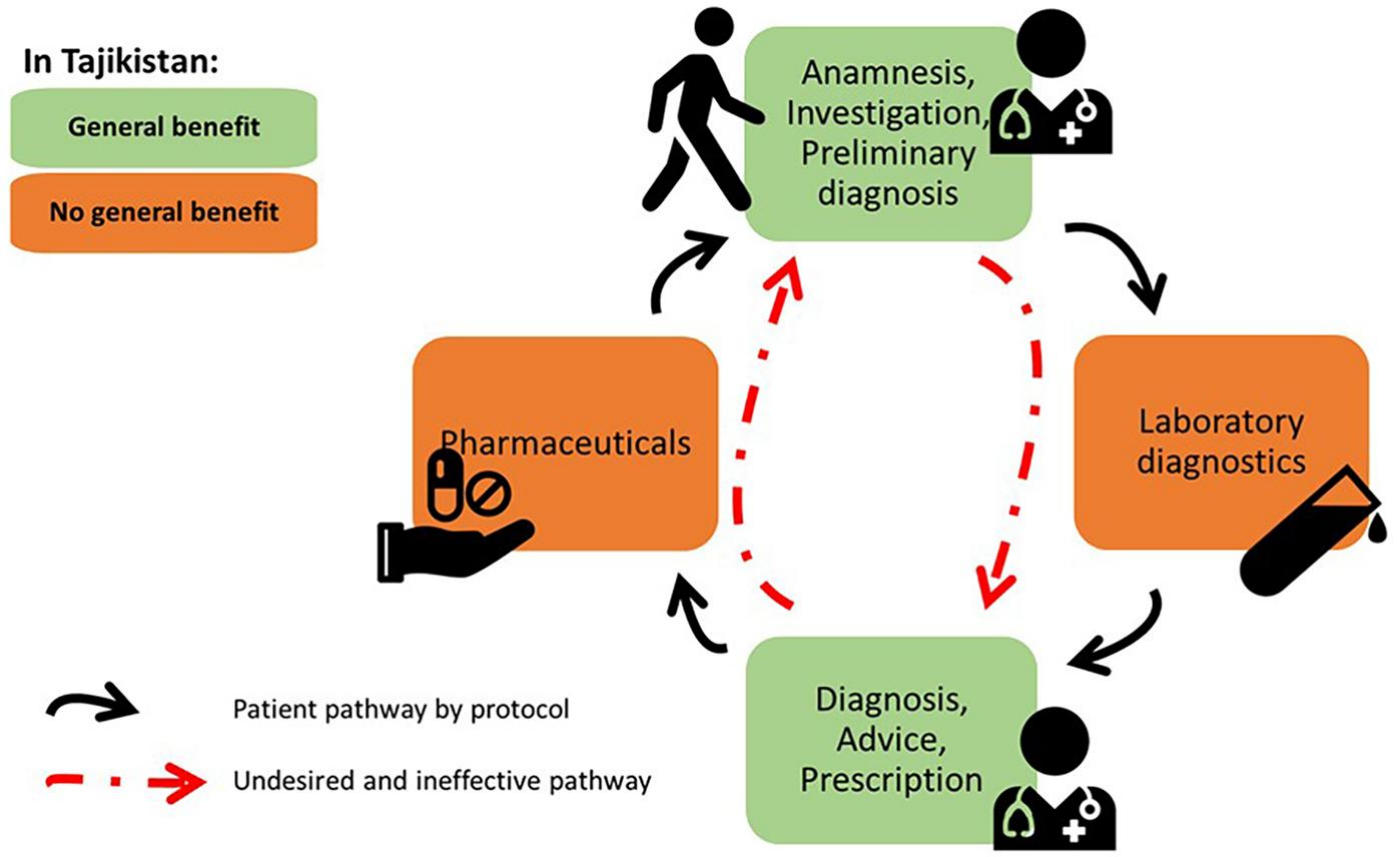
Ineffective chronic care due to gaps in the current BP in Tajikistan. Developed from concept presented in Wollum A., et al., (18).

Chronic NCDs are most effectively managed by a continuous, iterative process of well-defined interventions, including availability of essential medicines and diagnostics (17, 18). The network of primary care clinics in Tajikistan is extensive and services like weight and blood pressure measuring at consultation are provided to all. The MoHSP and international organizations have also made large efforts to implement and increase adherence to clinical guidelines. But the absence of laboratory diagnostics and outpatient medicines in the current BP interrupts the chain of effective disease management for patients who cannot pay, or opt not to pay, for these relatively simple elements of the care pathway (Figure 1).

The BP does include some point-of-care diagnostics, such as sugar level tests and urethral and vaginal smear tests for pregnant women. But these few items are not sufficient to diagnose and manage the most common NCDs and, in reality, access is limited and utilization is subject to out-of-pocket payments (11). Similarly, specific medicines are formally provided for free-of-charge distribution to beneficiary groups through vertically organized programs, such as insulin for diabetic type 1 patients by endocrinology centers and basic medicines for diarrhea and respiratory infections for children, but data on utilization is not available and both clinicians and patients testify to difficulties in affordable access.

### 3.2 Financial hardship caused by households' medicine expenses

In addition to forgone care causing illness and unnecessary later care needs, financial hardship caused by gaps in essential benefits is common. In 2022, 18% of Tajikistan households experienced catastrophic health spending, heavily concentrated among the poorest households. About 70% of all cases were caused by people's expenditure on outpatient medicines (19), although it's unknown how much of these are from the WHO Essential Medicines List (EML) and prescribed by protocol.

### 3.3 Cost-sharing rules drive utilization based on ability-to-pay rather than need

Public resources are spread thin across all types of specialized services, inpatient and outpatient, with heavy user-fees in practice being the main source of funding. here is an apparent risk that public resources are being spent on relatively wealthy and healthy individuals who can afford these user fees. In combination with the highly prevalent informal payments for health care, the user-fees risk that utilization of publicly provided services is based on who can pay, as opposed to who is in most need, leads to inequities and financial hardship.

The defined beneficiary groups are questionable from a BP perspective. For example, groups who typically have high health needs, such as children above one year, people aged under 80 years, and people with chronic diseases, do not fall within any of the beneficiary groups. Pediatric care tends to be beneficial when health gains and costs are compared (20). Both for social protection and cost-effectiveness reasons, neither 1 nor 80 are purposeful age thresholds. The age cut is also not aligned with other legislation, e.g., the 2008 Social Health Insurance law, which defines “child” at age 16 and is probably a more rational threshold.

## 4 Directions for a new generation of benefits package in Tajikistan

### 4.1 Introduce a criteria-based benefits package

An absence of clear priority setting rules and an overpromising as to what the state can provide risk leading to *implicit prioritizations* in all health systems, for example lower-than-necessary quality services, informal payments, or long waiting times. Criteria-based prioritization (6, 21) between specific conditions/diagnoses (horizontal prioritization) and between interventions within a specific condition/diagnosis (vertical prioritization) is common in countries with formalized health technology assessment processes (22) and are increasingly applied in middle-income contexts (23–25). Tajikistan's BP regulation and its implementation lacks explicit criteria for prioritization. In Supplementary File 2, we therefore suggest a set of criteria based on WHO guidelines (6) and objectives expressed in the national health strategy. An intervention is considered for inclusion in the BP if it addresses a condition prominent in the country’s burden of disease, is cost-effective, mitigates social vulnerability, and, finally, can be covered by the government’s health budget. The criteria are applied in a stepwise manner, with each criterion serving as a prerequisite for the next and only interventions that satisfy all criteria in a sequence being eligible for inclusion. For example, including interventions that the government budget can afford but which have less medical need (burden of disease) or are less effective relative to their cost (cost-effectiveness) than alternative interventions would mean priority setting is governed by budget space rather than medical needs.

### 4.2 Close the care cycle for prevalent chronic conditions with low-cost interventions

Effective detection and management of primary care–sensitive conditions require simple yet essential diagnostic tools. The WHO Essential Diagnostics List (EDL) recommends a basket of diagnostic tests that should be available at point-of-care and in laboratories and includes a designated section on primary-care-relevant tests (26). Next, effective control and treatment can come at a low cost. The annual pharmaceutical cost for controlling blood pressure of patients with uncomplicated hypertension in India has been estimated to be $2–4 per patient (27). Among adults aged 30-79 in Tajikistan, only 33% of those diagnosed with hypertension take their medicine (28). The individual's cost creates a barrier for some and signals that the public system does not find it important. A defined set of generic medicines for management of the largest chronic NCDs should be in the BP based on the WHO EML (29).

The challenge with both the EML and EDL from a BP perspective is that they do not prioritize among their specific items and hence do not rank what is most crucial to fund publicly within the lists. This has to be defined in each country separately, primarily depending on the budget situation (30, 31). Hence, these lists provided by the WHO can be seen as gross lists where all BP candidates for prescribed pharmaceuticals and diagnostic interventions are listed but with further prioritization needed. For example, the list of medicines can be short to start with, covering first-line options in the medical protocols for hypertension, diabetes, and asthma, and can be extended in updates of the BP. Both Uzbekistan and Ukraine are good examples of countries with earlier reforms where drug lists have been introduced on different levels of ambition, with later extensions based on market conditions and budget availability. Ukraine introduced a nation-wide list in 2018 and later extended the number of medicines (32). In Uzbekistan, a list was introduced in one region in 2022 and later extended geographically (33).

### 4.3 Abolish user fees for prioritized services

The currently applied user-fees for maternal and child care do not align well with either the priority setting criteria or the national health strategy. The next BP needs to expand free-of-charge services in this direction. Instead of 80% cost-sharing across all hospital services, they can be divided into two simple tiers, one with 0% cost-sharing (free-of-charge for the patient) and one with 100% (full cost paid by the patient), by type of services. Free-of-charge maternal and child services can be made budget neutral by charging other services with lower priority at their full cost. Supplementary File 3 illustrates this with hospital utilization and facility-level revenue data from the Tajikistan district of Tojikobod.

**Figure 2:**
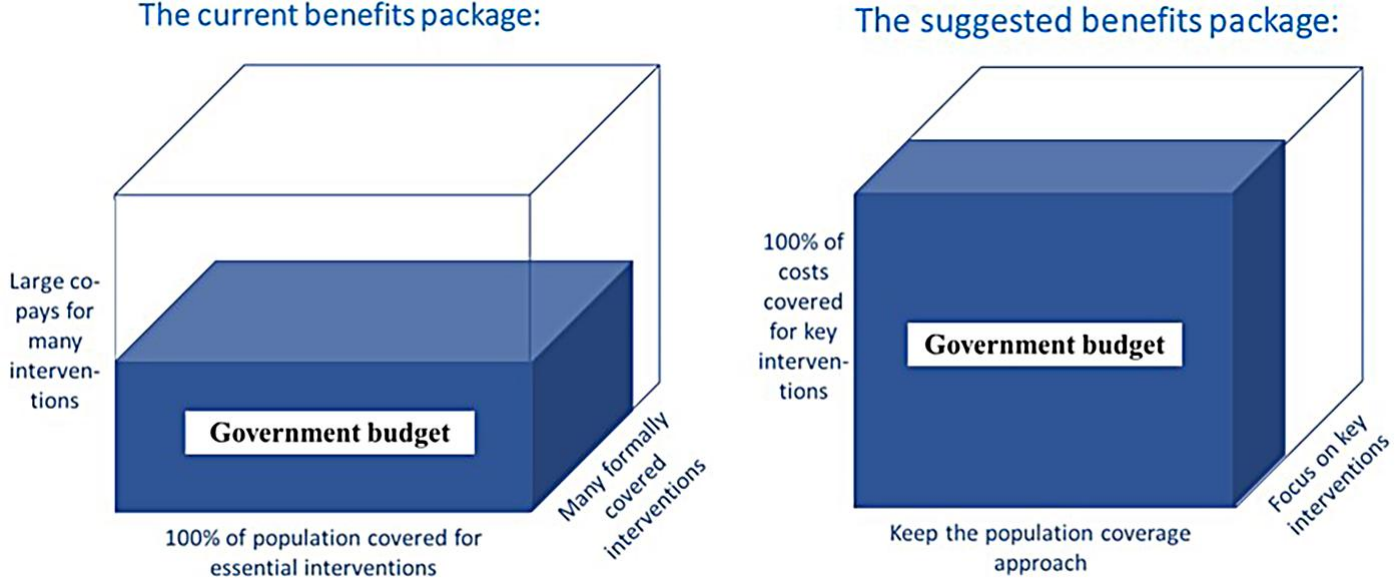
Illustration of the principal shift in interventions and co-payments suggested in this article. Developed from concept presented in World Health Report 2010: Health systems financing - the path to universal coverage (page 12) (5).

These suggested changes are illustrated in the three-dimensional UHC framework by population, interventions, and cost sharing (Figure 2). While the current population coverage definition is kept, the system would not “promise” the same extensive package of interventions but instead abolish user-fees on those with the highest priority.

## 5 Discussion

There are important implementation aspects of these suggestions to consider that are not covered by the scope of this article. But developing the new BP must not only be about describing a set of health care interventions and their possible user-fees but also about rules for its continuous development. It is impossible for any country to include all desired benefits at once. The task is to include the most essential benefits first and, by means of explicit prioritization, extend them when the budget allows. With time, the disease burden changes, new health interventions become available, and costs change. Countries with well-working BPs continuously manage this development with regular updates (34). This means the new generation of BP must not be seen as a static list but a continuously developing regulation. This continuous process requires defined roles for government institutions and rules of implementation, methods to apply the criteria, as well as institutional capacity with staff resources who consistently perform analytical work and draft concrete suggestions (35). There are related system challenges in need of development, among them the weak information system lacking individual-level data on health status and care utilization in the population. Implementation of a new benefits package must also relate to how user-fees are budgeted and used by clinics. The suggested changes will move funding flows within the system and, although overall budget effects can be made neutral and the public reimbursement system can mitigate effects for clinics, all stakeholders will not be affected equally.

## 6 Conclusions

This article shows how BP design can improve health system performance in a budget-constrained setting. The traditional approach in Tajikistan, in which budget priorities are defined by the number of facilities and staff, needs to shift to be purposefully focused on conditions and interventions with the most impact on health system objectives. Neighboring countries in Central Asia are doing exactly this, as exemplified by Uzbekistan where budget formulation is increasingly defined by priority health care as opposed to staff and physical resources (33).

## Data Availability

The data analyzed in this study is subject to the following licenses/restrictions: Unpublished aggregate (by district) health care utilization data have been used in the assessment. Requests to access these datasets should be directed to farrukhegamov@gmail.com.
