# Supplementary material for: Health care benefits package design to improve outcomes in resource-constrained settings: suggestions for Tajikistan
Source: Front Health Serv. 2025 Sep 16;5:1617679. doi: 10.3389/frhs.2025.1617679 (PMC12482916; doi:10.3389/frhs.2025.1617679)
Supplement: Supplementary file 1 [file Table1.docx]

**Supplement 1. PHC and outpatient specialist services included in the current benefits package**

Services provided free of charge for everyone include prevention, diagnostics, treatment and dental care.

- Prevention includes:
  - consultations and promotion of healthy lifestyle;
  - vaccination of children according to the national immunization calendar;
  - anonymous counselling on HIV/AIDS and sexually transmitted infections;
  - health check-ups for children aged under 5 years;
  - health check-ups of schoolchildren;
  - dispensary observation of diagnosed patients, with the exception of additional laboratory and instrumental diagnostics; and
  - targeted measures to prevent diseases.
- Diagnostics includes:
  - examination of the patient by a family doctor; and
  - basic laboratory diagnostics for pregnant women.
- Treatment includes:
  - urgent medical care;
  - immobilization;
  - prescribing medications and other types of treatment;
  - injections of medicines purchased by patients (intravenous, intramuscular, subcutaneous); and
  - medical interventions (according to a predefined list, which was supposed to be approved by the MoHSPP but has not yet been released).
- Dental care includes:
  - preventive examinations for children and pregnant women, twice per year;
  - emergency dental treatment; and
  - specialist dental care provided to vulnerable groups.

Extended services are available free of charge for vulnerable groups, and with 80%
co-payment for the general population. These include:

- specialist consultations, with a referral from a family doctor; and
- basic laboratory and diagnostic tests (basic blood screening, blood test for malaria, testing of donor blood for bloodborne infections, general urine analysis, microscopy of urethral and vaginal smear tests of pregnant women, sputum analysis, blood sugar testing (and urine), electrocardiography).

Source: The Order 600 of the Government of the Republic of Tajikistan *About the Procedure for rendering medical and sanitary services to citizens of the Republic of Tajikistan by organizations of the state health care system* is not published by the Government in English but available on

https://cis-legislation.com/document.fwx?rgn=84665
