# Supplementary material for: Health care benefits package design to improve outcomes in resource-constrained settings: suggestions for Tajikistan
Source: Front Health Serv. 2025 Sep 16;5:1617679. doi: 10.3389/frhs.2025.1617679 (PMC12482916; doi:10.3389/frhs.2025.1617679)
Supplement: Supplementary file 2 [file Table2.docx]

**Supplement 2. Suggested criteria for the new BP in Tajikistan, in order of application**

These four suggested criteria follow the approach as presented in Glassman et al.,^21^ which explains how a BP should be created based on consistent and transparent criteria that align with the health system's objectives. The criteria enable consistent prioritizing over time when contextual factors like budget or prices change, and allow health systems to establish agencies with clear roles for evaluating technologies and services.

1. **Burden of disease**

The BP must reflect the dominating burden of disease in Tajikistan and give priority to preventive services that address these. The BP must identify interventions that address this burden.

1. **Cost-Effectiveness of interventions available**

State budget health spending must not be decided in isolation by its medical effectiveness or its cost, but by how effective it is relative to its cost. This cost per effect ratio informs whether investing in one health care intervention is more or less beneficial than another.

1. **Affordability of care for the most vulnerable**

To mitigate social vulnerability, Tajikistan needs to decrease financial hardship due to health care needs.

1. **Budget impact**

All interventions in the BP must be realistically funded to avoid implicit priorities in clinical settings. However, a key element in rational use of resources is to assess the budget impact of any item in the BP only after other criteria are assessed to avoid priorities guided by budget space rather than health needs.
