# Supplementary material for: Health care benefits package design to improve outcomes in resource-constrained settings: suggestions for Tajikistan
Source: Front Health Serv. 2025 Sep 16;5:1617679. doi: 10.3389/frhs.2025.1617679 (PMC12482916; doi:10.3389/frhs.2025.1617679)
Supplement: Supplementary file 3 [file Table3.docx]

**Supplement 3. The budget effect of free‑of‑charge maternal and child services can be neutral**

The table describes the effect from a co‑payment collection perspective in the Tojikobod district (51 000 population), based on the official co‑payment price list. A total of 597 interventions were made in the district hospital in 2023. By shifting co‑payment revenue from maternal and child services to adult services, the budget implication is practically zero (compare last row).

**Facility revenue by the current BP (80% co‑payment) and an alternative 0% and 100% by differentiated groups of services.**

| Classification by interventions, as practiced in the current benefit package. | Average full cost, somoni^1^ | Volume, hospital services^2^ | Current  80%  co‑pay | **Total hospital revenue^3^** | Alternative co‑pay levels  (0% and 100%) | **Hospital**  **revenue at 0% and 100% co‑pays^3^** |
| --- | --- | --- | --- | --- | --- | --- |
| Adult surgery  (general/abdominal) | 540/760 | 299 | 432/608 | **122 147** | 540/760 | **178 230** |
| Child surgery  (general/abdominal) | 420/700 | 95 | 336/560 | **34 986** | 0 | **0** |
| Adult Therapy | 500 | 26 | 400 | **7 488** | 500 | **10 926** |
| Child Therapy | 440 | 79 | 352 | **18 287** | 0 | **0** |
| Obstetric surgery/Delivery | 500/320 | 85 | 400/256 | **5 644** | 0 | **0** |
| Gynecology | 450 | 13 | 360 | **1 146** | 0 | **0** |
| Total hospital revenue from user fees |  | | | **189 698** |  | **189 151** |

1) Official price as applied nationally 2023

2) Tojikobod district

3) Estimates based on volumes, official price, and applied national averages for payment exemption (28% poor population, 2/3 of women following the national antenatal care protocol).

Source: Author’s calculation based on data from the Ministry of Health and Social Protection of the Republic of Tajikistan, collected through the Statistical medical form #30
